# Principles of Neurorehabilitation After Stroke Based on Motor Learning and Brain Plasticity Mechanisms

**DOI:** 10.3389/fnsys.2019.00074

**Published:** 2019-12-17

**Authors:** Martina Maier, Belén Rubio Ballester, Paul F. M. J. Verschure

**Affiliations:** ^1^Laboratory of Synthetic, Perceptive, Emotive and Cognitive Systems, Institute for Bioengineering of Catalonia, The Barcelona Institute of Science and Technology, Barcelona, Spain; ^2^Institucio Catalana de Recerca I Estudis Avançats, Barcelona, Spain

**Keywords:** neurorehabilitation, motor learning, plasticity, stroke, principles

## Abstract

What are the principles underlying effective neurorehabilitation? The aim of neurorehabilitation is to exploit interventions based on human and animal studies about learning and adaptation, as well as to show that the activation of experience-dependent neuronal plasticity augments functional recovery after stroke. Instead of teaching compensatory strategies that do not reduce impairment but allow the patient to return home as soon as possible, functional recovery might be more sustainable as it ensures a long-term reduction in impairment and an improvement in quality of life. At the same time, neurorehabilitation permits the scientific community to collect valuable data, which allows inferring about the principles of brain organization. Hence neuroscience sheds light on the mechanisms of learning new functions or relearning lost ones. However, current rehabilitation methods lack the exact operationalization of evidence gained from skill learning literature, leading to an urgent need to bridge motor learning theory and present clinical work in order to identify a set of ingredients and practical applications that could guide future interventions. This work aims to unify the neuroscientific literature relevant to the recovery process and rehabilitation practice in order to provide a synthesis of the principles that constitute an effective neurorehabilitation approach. Previous attempts to achieve this goal either focused on a subset of principles or did not link clinical application to the principles of motor learning and recovery. We identified 15 principles of motor learning based on existing literature: massed practice, spaced practice, dosage, task-specific practice, goal-oriented practice, variable practice, increasing difficulty, multisensory stimulation, rhythmic cueing, explicit feedback/knowledge of results, implicit feedback/knowledge of performance, modulate effector selection, action observation/embodied practice, motor imagery, and social interaction. We comment on trials that successfully implemented these principles and report evidence from experiments with healthy individuals as well as clinical work.

## Introduction

So far there is no clear understanding of the principles underlying effective neurorehabilitation approaches. Therapeutic protocols can be readily described by the following aspects: the body part trained (e.g., the legs), the tools or machines used for the training (e.g., a treadmill), the activity performed (e.g., walking), and when the therapy commences (e.g., during the acute phase after a stroke). However, an intervention typically includes more elements. For instance, the use of the less affected limb can be restricted, and the therapist can encourage the patient to spend more time exercising or give feedback about task performance. While some interventions, like CIMT, clearly define their active ingredients (Carter et al., 2010; Proffitt and Lange, 2015) that should lead to effective recovery (Kwakkel et al., 2015), most others do not. Neurorehabilitation research aims to find interventions that promote recovery and to establish whether the presence or absence of improvement can be explained by any neuronal changes that occur in the post-stroke brain (Dobkin, 2005). Neuroscience can help us to create interventions that lead to changes in the brain; however, with no clear understanding of what an intervention does, attributing causality remains difficult. One way to formalize an intervention is by breaking it into parts, studying the behavioral and neural effects of these parts, and deriving principles from them–in the case of stroke neurorehabilitation, these would be principles that optimize acquisition, retention, and generalization of skills.

While there are plenty of meta-analyses that look at training effectiveness in terms of individual body parts/functions, tools, or machines and activities (Langhorne et al., 2009; Veerbeek et al., 2014), the effect of experience remains much less clear in spite of attempts to formalize and identify the principles of neurorehabilitation. A review of principles of experience-dependent neural plasticity by Kleim and Jones (2008) explains why training is crucial for recovery. According to their work, neurorehabilitation presumes that exposure to specific training experiences leads to improvement of impairment by activating neural plasticity mechanisms. Consequently most of the work in the field focuses on the identification of scientifically grounded principles that should guide the design of these training experiences. In this vein, Kleim and Jones (2008) elaborated on five main principles of effective training experience — specificity, repetition, intensity, time, and salience — but offered little concrete applicability. Another synthesis addressed further principles (forced use, massed practice, spaced practice, task-oriented functional training, randomized training); however, the main focus of the review was on individual body functions, methods, or tools, providing a global view on rehabilitation strategies (Dobkin, 2004). Two meta-analyses investigated specific principles. One looked only at the principle of intensity and found that more therapy time did enhance functional recovery (Kwakkel, 2009). Another determined that repetition does improve upper and lower limb function (Thomas et al., 2017). However, both studies did not investigate the mechanisms that would lead to the effects observed. Similarly, a review that analyzed CIMT, which combines several principles in one method, gained interesting insights in its efficacy but did not explain the results from a neuroscientific, mechanistic point of view (Kwakkel et al., 2015). The work by Levin et al. (2015), on the other hand, tried to link the principles of motor learning to the application of these principles in novel rehabilitation methods while offering some neuroscientific reasoning for doing so. Their review addresses the difficulty of the task, the organization of movement, movements to the contralateral workspace, visual cues and objects and the interaction with them, sensory feedback, feedback about performance and results, repetitions, variability, and motivation. However, the included motor control and motor learning principles were not well defined and therefore leave room for interpretation (Levin et al., 2015).

In a previous meta-analysis (Maier et al., 2019), we compiled a list of principles for neurorehabilitation based on literature on motor learning and recovery: massed practice, dosage, structured practice, task-specific practice, variable practice, multisensory stimulation, increasing difficulty, explicit feedback/knowledge of results, implicit feedback/knowledge of performance, movement representation, and promotion of the use of the affected limb. We then performed a content analysis to determine whether these principles were present in the clinical studies included in the review, but we did not provide an analysis of the principles identified. In this work, we aim to extend the number of principles found and, for each of them, unify the neuroscientific literature from human or animal studies on motor learning and comment on the observed neuronal effects. We also include evidence from clinical studies to show its effect in recovering functionality after stroke. Some principles already serve as building blocks of effective rehabilitation programs, e.g., CIMT (Kwakkel et al., 2015), Bobath (Kollen et al., 2009), enriched rehabilitation (Livingston-Thomas et al., 2016), VR-based rehabilitation (Laver et al., 2017), and exogenous or robotic interventions (Langhorne et al., 2011). However, transferring these principles into clinical practice faces the challenge of operationalizing them. We comment on these difficulties and the gaps between theory, evidence, and operationalization that we encountered. Consequently, this work can serve clinicians and researchers as a practical guide of principles to investigate further effective neurorehabilitation approaches.

## Materials and Methods

In this conceptual analysis, the rehabilitation experience is broken down into individual parts that are termed principles of neurorehabilitation. They are principles because they are evidenced by experimental data, and together, they could form the foundation of a higher-order theoretical framework. As a first attempt, a list of 11 principles was compiled based on existing literature in a recent meta-analysis on the effectiveness of VR-based rehabilitation systems for stroke recovery (Maier et al., 2019). For the current work, the list has been revised, and additional principles have been identified through a computerized search in PubMed Central using the keywords “principles of motor learning,” “principles of recovery,” “principles of experience-dependent learning” and “principles of neurorehabilitation.” We restricted the search to the last 5 years to obtain currently used principles. We focused on reviews, perspectives, and debates around rehabilitation methods and interventions for stroke recovery and excluded articles that explained study protocols or clinical trials, prevention methods, pharmaceutical or medical interventions, or stroke taxonomies. The principles mentioned in each paper were compared with the original list and added if they were not present. Afterward, we summarized for each principle the historical background based on motor learning literature and its contribution to learning based on human or animal studies. Further, where available, neurological effects and clinical outcomes were included as well.

## Identification of Principles of Neurorehabilitation

Our computerized search yielded 548 records, of which 74 were deemed adequate for further screening after we examined if their titles either contained any of the search terms or appeared to discuss post-stroke rehabilitation strategies. After analysis of their abstracts and full-texts, the principles mentioned in 17 articles were extracted. We excluded papers if their title or abstract reported or compared surgical or pharmaceutical interventions as well as if they discussed stroke taxonomies, proposed study protocols or clinical trials, covered principles unrelated to stroke and/or stroke rehabilitation itself (e.g., principles for disease prevention, pre- and post-operative care, care facilities, patient management, therapist education, nursing practice, dietary recommendation, veterinary etc.), or looked into patient or caregiver perception. The articles and reviews selected spawned various research fields in neurorehabilitation: Motor learning (Winstein et al., 2014), therapies [physical therapy (Veerbeek et al., 2014), upper limb immobilization (Furlan et al., 2016), environmental enrichment (Livingston-Thomas et al., 2016), aerobic training (Billinger, 2015; Hasan et al., 2016), CIMT (Kwakkel et al., 2015; Zhang et al., 2017), cognitive rehabilitation (Middleton and Schwartz, 2012), music therapy (Zhang et al., 2016)], tools and methods [hand robotics (Yue et al., 2017), VR (Darekar et al., 2015; Fu et al., 2015), neurofeedback (Renton et al., 2017)], and principles [dose and timing (Basso and Lang, 2017)]. Together with previously collated literature, we identified 15 principles.

The identified principles from the meta-analysis are as follows:

• Massed practice/repetitive practice (Middleton and Schwartz, 2012; Veerbeek et al., 2014; Fu et al., 2015; Kwakkel et al., 2015; Furlan et al., 2016; Zhang et al., 2016).
• Spaced practice (Middleton and Schwartz, 2012; Billinger, 2015; Hasan et al., 2016; Livingston-Thomas et al., 2016).
• Dosage/duration (Veerbeek et al., 2014; Winstein et al., 2014; Billinger, 2015; Darekar et al., 2015; Kwakkel et al., 2015; Hasan et al., 2016; Livingston-Thomas et al., 2016; Basso and Lang, 2017; Zhang et al., 2017).
• Task-specific practice (Veerbeek et al., 2014; Winstein et al., 2014; Fu et al., 2015; Kwakkel et al., 2015; Furlan et al., 2016; Livingston-Thomas et al., 2016; Yue et al., 2017).
• Variable practice (Darekar et al., 2015; Fu et al., 2015; Livingston-Thomas et al., 2016).
• Increasing difficulty (Winstein et al., 2014; Fu et al., 2015; Kwakkel et al., 2015; Furlan et al., 2016; Hasan et al., 2016; Livingston-Thomas et al., 2016; Zhang et al., 2017).
• Multisensory stimulation (Veerbeek et al., 2014; Livingston-Thomas et al., 2016; Yue et al., 2017).
• Explicit feedback/knowledge of results (Middleton and Schwartz, 2012; Veerbeek et al., 2014; Darekar et al., 2015; Fu et al., 2015; Renton et al., 2017).
• Implicit feedback/knowledge of performance (Veerbeek et al., 2014; Darekar et al., 2015; Fu et al., 2015; Zhang et al., 2016; Renton et al., 2017; Yue et al., 2017).
• Modulate effector selection (Veerbeek et al., 2014; Winstein et al., 2014; Kwakkel et al., 2015; Furlan et al., 2016; Zhang et al., 2017).
• Action observation/embodied practice (Veerbeek et al., 2014; Fu et al., 2015; Yue et al., 2017).

Additional principles encountered through the search:

• Goal-oriented practice (Winstein et al., 2014; Fu et al., 2015; Yue et al., 2017).
• Rhythmic cueing (Middleton and Schwartz, 2012; Veerbeek et al., 2014; Zhang et al., 2016).
• Motor imagery/mental practice (Veerbeek et al., 2014).
• Social interaction (Winstein et al., 2014; Fu et al., 2015; Livingston-Thomas et al., 2016; Zhang et al., 2017).

In the following sections, we summarize for each principle the theoretical background, the evidence for motor learning, and the clinical effectiveness. We also added studies that comment on the neurological changes observed after applying the principles in motor learning tasks. The detailed neurological changes reported by these studies can be found in Table 1.

**TABLE 1:** Overview of the neuronal changes due to exposure to principles of neurorehabilitation included in this manuscript.

			
**Experience-dependent changes**	**Principles**	**Brain areas**	**References**
			
**Cellular/neuronal level**			
Increased neuronal activity	Spaced practice	Task/stimulus-dependent	Gerbier and Toppino, 2015
Increased cell survival and improved LTP	Spaced practice	Hippocampus	Scharf et al., 2002; Sisti et al., 2007
Upregulation of growth factors (protein 43, synaptophysin)	Dosage	Intact corticospinal tract	Zhao et al., 2013
Inhibition of upregulation of growth-inhibiting factors (NogoA, Nogo receptors and RhoA)	Dosage	Peri-infarct cortex	Zhao et al., 2013
Dopamine-dependent synaptic plasticity	Explicit feedback	Striatum	Kawagoe et al., 1998
Complex spikes in Purkinje cells	Implicit feedback	Cerebellum	Kitazawa et al., 1998

**Cortical motor areas**			
Expansion or change of effector representation/cortical map, dependent on effector trained	Massed practice	Motor cortex	Plautz et al., 2000
Increased excitability	- Dosage	- Motor cortex	- Liepert et al., 2000; Veerbeek et al., 2014
	- Variable practice	- Motor cortex	- Lage et al., 2015; Lin et al., 2011
Normalization of activation in ipsilesional cortex	Dosage	Motor cortex	Schaechter, 2004
Change in sensorimotor organization	Multisensory stimulation	Motor cortex	Rosenkranz and Rothwell, 2006
Increased neuronal recruitment during acquisition, decreased activity during retention	Variable practice	Prefrontal areas, PMA, inferior frontal areas	Lage et al., 2015; Lin et al., 2011
Increased cortical activation in lesioned hemisphere during paretic movement	- Task-specific practice	- SMC, PMC - SMC	- Jang et al., 2003 - Wilkins et al., 2017
	- Modulate effector selection	- SSC/SMA, dorsal PMC	- Johansen-Berg et al., 2002
Increased cortical activation in contralesional hemisphere during paretic movement	Rhythmic cueing	SMC	Luft et al., 2004
Decreased activation in contralesional hemisphere during paretic movement	Task-specific practice	- SMC, PMC, SMA - Motor cortex - SMA, PMA	- Jang et al., 2003 - Boyd et al., 2010 - Wilkins et al., 2017
Increased laterality index during paretic movement	Task-specific practice	- SMC - Motor cortex - SMC, SMA, PMA	- Jang et al., 2003 - Boyd et al., 2010 - Wilkins et al., 2017
Increased power spectra	Multisensory stimulation	SMC, SSC	Gomez-Rodriguez et al., 2011

**Fronto-parietal network**			
Increased activation of contralateral fronto-parietal network	Goal-oriented practice	Motor cortex, SMA, SSC, parietal areas	Nathan et al., 2012
Increased activation of bilateral parietal areas, together with lateralized pre-motor areas and sensorimotor areas	Increasing difficulty	PMC, SMA, SMC, SPA, IPA	Wexler et al., 1997; Winstein et al., 1997
Increased activation of bilateral parietal, premotor and visual areas	Action observation	Dorsal and ventral PMC, pre-SMA, SPA, IPA, visual cortex	Hardwick et al., 2018
Increased activation of lateralized parietal areas, together with pre-motor areas	Motor imagery	Bilateral dorsal PMC, left ventral PMC, Bilateral pre-SMA, left IPA, left SPA,	Hardwick et al., 2018
Increased activation and functional connectivity	Mirror therapy	- Ipsilateral motor cortex, visual processing areas	- Arya, 2016
		- Bilateral PMA, contralateral SMA and SMC, parietal cortex	- Hardwick et al., 2018

**Cerebellum**			
Increased activation	- Rhythmic cueing	- Cerebellum (ipsilesional)	- Luft et al., 2004
	- Modulate effector selection	- Cerebellum (bilateral)	- Johansen-Berg et al., 2002

**Somatosensory Cortex**			
Reversal of SEP to pre-infarct	Dosage	Somatosensory cortex	Joo et al., 2012

**Extended networks**			
Auditory feedback lead to reduced activity during acquisition	Implicit feedback	SMC, SMA, opercular, temporal and parietal areas	Ronsse et al., 2011
Visual feedback lead to increased activity during acquisition	Implicit feedback	Occipital gyri, cerebellar lobules and vermis	Ronsse et al., 2011
Visual feedback preserved activation, when no feedback was given during testing	Implicit feedback	Occipitotemporal cortex	Ronsse et al., 2011
Auditory feedback suppressed activity, when no feedback was given during testing	Implicit feedback	Auditory cortex	Ronsse et al., 2011
Increased fractional anisotropy	Rhythmic cueing	Arcuate fasciculus (white matter tract connecting auditory and motor regions)	Moore et al., 2017
Activity in social cue network	Social interaction	Right posterior STS, right anterior STS, right TPJ	Redcay et al., 2010

*LTP, long-term potentiation; PMC, premotor cortex; SPA, superior parietal area; IPA, inferior parietal area; SEP, somatosensory-evoked potentials; SMC, sensorimotor cortex; SMA, supplementary motor area; SSC, somatosensory cortex; STS superior temporal sulcus; TPJ, temporoparietal junction.*

### Massed Practice/Repetitive Practice

Massed practice was defined as work episodes with very brief to no rest periods (Schmidt and Lee, 2011). Within a work episode, a skill can be trained repeatedly in a *constant* or *blocked* fashion (Ammons, 1947; Mulligan et al., 1980). In the field of rehabilitation, the term describes the prolonged and repeated use of the more affected limb (Taub et al., 1999). Theoretically, learning through repetitions can speed-up the shaping of priors, which, together with likelihoods based on sensory input, aid in making an optimal estimate for action selection (Körding and Wolpert, 2006). Animal studies have shown that repeating skilled movements leads to localized changes in the area responsible for the movement, whereas the pure repetition of unskilled movement does not (Plautz et al., 2000). In humans, early studies have shown that blocked practice leads to faster acquisition, but poorer retention and less transfer than variable practice (Shea and Morgan, 1979) and that massed practice without breaks seems less effective for motor performance (Ammons, 1947; Ammons and Willig, 1956).

In standard therapies or clinical studies, the amount of repetition is typically not quantified but was observed to be an order of magnitude lower than in studies investigating recovery in rats and monkeys (Lang et al., 2007). Instead, the evidence for massed practice relies typically on the number of sessions or duration (French et al., 2016). A study looking into the feasibility of translating repetition amounts of animals to humans found improved motor functioning after training with high-repetition doses. However, no “pure” repetition training was provided, as the protocol included a variety of tasks that increased in difficulty (Birkenmeier et al., 2010). On the contrary, a study comparing four groups with different repetition amounts did not find significant differences based on the number of repetitions (Basso and Lang, 2017). This intervention included other principles as well. Meta-analyses confirm the mixed effects of repetitive training on improvement (Langhorne et al., 2011; Veerbeek et al., 2014; French et al., 2016; Thomas et al., 2017). Hence, massed practice appears to be a commonly used ingredient, but its clinical operationalization is often confounded with other principles. In order to investigate its true effects on recovery and compare across studies, the repetitions within a training session and across therapy duration should be measured and quantified.

### Spaced Practice

Spaced practice implies that training should be structured in time to include rest periods between repetitions or sessions (Lee and Genovese, 1988; Schmidt and Lee, 2011). Instead of spaced practice, the term *distributed practice* is often used in literature. However, some authors use the term distributed practice as a combination of spaced and massed practice (Cepeda et al., 2006). Research on human skill acquisition suggests that increasing the time spacing between learning periods improves final test performance (Cepeda et al., 2006). However, when these learning periods are too long, learning and retention rates drop (Savion-Lemieux and Penhune, 2005). The mechanisms behind the effects of distributed practice remain unclear. It has been hypothesized that the first exposure to a stimulus pre-activates its representation in memory, requiring no further activation in a subsequent repetition trial, leading to a poorer internal representation of that stimulus, which has been termed as the repetition suppression effect (Gerbier and Toppino, 2015). Animal and fMRI studies support this hypothesis, showing that neuronal activation decreases after stimulus repetition where the magnitude is modulated by the delay between the first and second presentation, with larger delays leading to greater decreases (Brown et al., 1987; Henson et al., 2000, 2004; Henson, 2003). Spaced practice might counteract the repetition suppression effect by canceling stimulus priming (Gerbier and Toppino, 2015). TMS revealed that primary and supplementary motor areas are involved in motor memory consolidation (Censor and Cohen, 2011), which might be facilitated by spaced practice. Further, learning and physical activity have been linked to hippocampal neurogenesis (Praag et al., 1999). Animal studies also suggest that spaced practice facilitates long-term memory formation (Okamoto et al., 2011; Yamazaki et al., 2015) by fostering the survival of cells in the dentate gyrus that are important for learning and memory (Sisti et al., 2007). Also, *in vivo* spacing of electrical stimulation facilitates the recruitment of protein-synthesis-dependent processes, which facilitates late LTP effects (Scharf et al., 2002; Gerbier and Toppino, 2015).

In the clinical field, only a few studies have investigated the effect of spacing on post-stroke recovery. A clinical study that investigated whether a CIMT protocol could be distributed over more days with less therapy time per day showed improvement in motor outcomes that were similar to previous CIMT protocols and superior outcomes in long-term quality of life (Dettmers et al., 2005).

### Dosage

Unlike in pharmacology, dosage is an ill-defined term in rehabilitation (Dobkin, 2005; Kwakkel, 2009). Generally, it is operationalized as the number of hours spent in therapy (Kwakkel, 2009; Birkenmeier et al., 2010; Veerbeek et al., 2014; Basso and Lang, 2017), the frequency of training sessions and the duration of a session (Dobkin, 2005), or the training amount required to stimulate learning (Wadden et al., 2017). High dosages are often equated with high intensity of training (Kwakkel et al., 2015). However, the intensity of training could also be operationalized as the metabolic cost, work rate, or perceived intensity through exertion (Billinger, 2015; Hasan et al., 2016), which are rarely measured in standard therapies except in fitness and aerobic protocols (Kwakkel, 2009).

Typically, inpatients receive only 22 (Veerbeek et al., 2014) to 60 min of training a day, with fewer minutes at later stages (Schaechter, 2004). There is some evidence that increasing therapy hours would be beneficial to speeding up functional recovery (Lohse et al., 2014; Veerbeek et al., 2014). At least 16 h of extra training (e.g., 71 more minutes per day for 3 months) within the first 6 months seem to be required for functional gains (Kwakkel et al., 2004; Veerbeek et al., 2014). However, there is some controversy over the benefits of increased training early after stroke (Schaechter, 2004; Dromerick et al., 2009; Kwakkel, 2009), and a pooled analysis revealed no evidence of an effect of additional doses (Hayward et al., 2014). Hence, the exact dose-response for different therapies at different stages post-stroke needs to be determined (Kwakkel, 2009; Basso and Lang, 2017). Also, it seems that motor performance needs to reach an asymptotic level in the first session to facilitate delayed performance gains across sessions or days. Therefore, delayed performance gains seem not to depend on repetition or over-night consolidation, but on the amount of training that induces asymptote in the individual’s performance (Hauptmann et al., 2005). Neurologically, high-dose rehabilitation protocols with extended training hours possibly induce structural plastic changes as well as a reorganization of neural networks (summarized by Kwakkel et al., 2015), increase cortical excitability and improve motor function and use (Liepert et al., 2000; Veerbeek et al., 2014). Several studies observed a normalization in ipsilesional cortex activity, which could underlie the functional gains (Schaechter, 2004).

### Task-Specific Practice

Task-specific practice postulates that changing the conditions of a task might require a change in the abilities needed to execute it; conditions during training should match the conditions during testing (Schmidt and Lee, 2011). Thus, the specific conditions of practice shape the internal sensorimotor representation of the skill learned (Nudo et al., 1996; Ridderinkhof et al., 2004), leading potentially to highly specialized skills (Keetch et al., 2005) whose performance is superior in transfer tasks that meet the training conditions (Schmidt and Lee, 2011). Grounded in this principle, conventional rehabilitation protocols focus their training on the execution of ADL, as they are deemed meaningful to the patient (Hubbard et al., 2009). Since the main target of rehabilitation is to enable the patient to perform ADL independently (Winstein et al., 2014), therapy might not prioritize the restoration of pre-stroke movement patterns but allows the patient to acquire compensatory movement skills.

One study with a large sample size found that task-specific practice appears to be similar to standard therapy in improving motor functionality (Winstein et al., 2016). On the other hand, smaller fMRI studies found that task-specific training facilitated motor learning and retention (Boyd et al., 2010) and induced a change in the laterality index, which was confirmed in other studies as well (Jang et al., 2003; Wilkins et al., 2017). However, while two studies found reduced activity in the contralesional cortex, one (Jang et al., 2003) found changes in neuronal activity patterns in both hemispheres. A study with TMS demonstrated a trend toward reduced interhemispheric inhibition following task-specific training (Singer et al., 2013).

### Goal-Oriented Practice

Since a given goal (e.g., throwing a ball into the basket) could be accomplished by many different motor synergies, it is assumed that movement control is achieved through the coupling of goal-specific functional movements. Goal-oriented practice, therefore, does not emphasize primarily individual muscles or movement patterns involved in execution but requires the patient to explore the couplings that are suitable to achieve the task (Horak, 1991). In general, motor skill performance and learning are enhanced if attention is directed to the effect of movement instead to the movement itself (Wulf and Prinz, 2001). Goal-oriented movements appear to produce a better reaching performance than the same movements without a goal (Wu et al., 2000), and setting specific, difficult goals leads to higher motor learning performance than non-specific goals (Gauggel and Fischer, 2001). It appears that probing a skill in a goal-directed fashion after overnight consolidation promotes better performance than probing the skill by drawing attention to finger movements (Cohen et al., 2005). Evidence from studies looking into tool-use in animals and humans suggest that, neurologically, action goals are represented as effector-dependent in the anterior intraparietal sulcus and primary motor areas, and as effector-independent in the ventral intraparietal sulcus and premotor cortex (Gallivan and Culham, 2015). Goal-oriented movements produce higher activity in sensorimotor areas (Nathan et al., 2012).

There is some evidence that goal-oriented practice is beneficial for recovery (Bosch et al., 2014). However, the described interventions seem to be confounded by other principles that are sometimes ascribed to goal-oriented training (Harvey, 2009).

### Variable Practice

Variable practice can be achieved in two ways: (1) by providing variability within a training sequence, a method termed as *variability of practice* (Schmidt, 1975), or (2) by randomizing the presentation of individual training sequences, a method termed as *random practice* or *contextual interference* (Battig, 1966; Shea and Morgan, 1979). Both methods have been shown to lead to better retention (Shea and Kohl, 1991) and enhanced generalization to similar but untrained tasks (McCracken and Stelmach, 1977) or movements (Shea and Morgan, 1979; Mulder and Hochstenbach, 2001; Park et al., 2016), despite hampering initial performance (Shea and Morgan, 1979). However, a random presentation of information might be detrimental to motor learning (Mulder and Hochstenbach, 2001). Imaging studies have shed some light on the mechanisms supporting these effects. fMRI and TMS studies in humans indicate that improved performance due to variable practice correlates with increased neuronal activity and connectivity in the areas of the motor learning network during acquisition, which is associated with better performance at retention stages (Lage et al., 2015). Also, the motor cortex showed greater excitability during retention. These results point to more efficient retrieval of motor memory due to variable practice (Lin et al., 2011). More complex bimanual visuomotor tasks that were practiced randomly have shown modality-specific activation patterns that led to the recruitment of areas related to visual processing (Pauwels et al., 2018). The effect of variable practice might be related to the strong link between the neuromodulatory systems that control neuronal plasticity and novelty, for instance, the dopaminergic (Redgrave and Gurney, 2006), cholinergic (Hasselmo et al., 1996), and noradrenergic systems (Vankov et al., 1995), which are used by the brainstem activation system for controlling the global state of arousal (Gur et al., 2007).

In the clinical context, one study that investigated random versus blocked practice failed to find an effect (Hayward et al., 2014). It seems that this principle is rarely studied explicitly in clinical studies (Darekar et al., 2015; Nielsen et al., 2015), but instead applied in conjunction with other principles to overcome boredom (Birkenmeier et al., 2010).

### Increasing Difficulty

According to Guadagnoli and Lee (2004) and based on the ideas from Marteniuk (1976), task difficulty can be described by the training requirements and conditions that are pertinent to the task, called the nominal task difficulty, and by how challenging the training is relative to the skill of the performer, called the functional task difficulty. Practice leads to fewer prediction errors and less need to process error information. Increasing the nominal task difficulty hence increases prediction errors and error processing demands. The optimal challenge point lies where functional task difficulty leads to a balance between information processing demands and performance, which is optimal for learning (Marteniuk, 1976; Guadagnoli and Lee, 2004). It has been shown that training with difficulty levels personalized to the learner’s capabilities leads to superior learning outcomes than when increases in difficulty are fixed (Wickens et al., 2013). Further, if subjects can control the task difficulty by themselves, their motor performance during acquisition and retention is significantly better (Andrieux et al., 2012). However, if difficulty surpasses one’s perceived ability to succeed, it might lead to detrimental effects on performance (Gendolla, 1999). Brain imaging studies showed increased activity in lateralized pre-motor and sensorimotor areas, but with an even more pronounced increase in parietal areas, pointing to a specialization of that area for task complexity (Wexler et al., 1997; Winstein et al., 1997). Potentially, noradrenergic neurons keep track of high or low task performance due to difficulty by switching their activity pattern preceding behavior (Rajkowski et al., 2004; Aston-Jones and Cohen, 2005).

In stroke rehabilitation, task difficulty has been partly investigated through *shaping* or *graded practice*. Shaping is a concept that was initially used by behaviorists studying operant conditioning in animals and that was successfully transferred from animals to humans by making it part of CIMT (Taub, 1976; Taub and Uswatte, 2003): The use of the impaired limb is augmented by progressively increasing the complexity of the required movement (Taub et al., 1994; Kwakkel et al., 2015). Although shaping appears to be one of the essential components of CIMT, its particular effect on motor recovery has not been studied on its own (Kwakkel et al., 2015). Increasing difficulty has been successfully used in standard care studies (Woldag et al., 2010), robot-assisted therapy (Lucca, 2009), and VR-based systems (Cameirão et al., 2012; Ballester Rubio et al., 2016), all of which showed beneficial effects on motor recovery. Task difficulty appears to be implicitly present in many tasks that investigate motor learning without being explicitly operationalized.

### Multisensory Stimulation

The perception and integration of multiple senses are fundamental abilities of the brain. Because sensory information is noisy, the integration of various modalities requires probabilistic estimations to enhance perception (Knill and Pouget, 2004). Studies in the cat superior colliculus showed that a single neuron could be responsive to several sensory modalities (Meredith and Stein, 1986; Wallace and Stein, 1996). In primates, the classic areas associated with multisensory processing are the superior temporal sulcus, the intraparietal cortex, and the frontal cortex, with newer studies confirming multisensory processing also in areas that were previously thought to be mainly unisensory (Ghazanfar and Schroeder, 2006). One sensory input (e.g., touch) can influence how another sensory modality is perceived (e.g., vision) (Driver and Noesselt, 2008); therefore, exposure to multisensory feedback can enhance the ability to detect, discriminate and recognize sensory information (Driver and Noesselt, 2008; Shams and Seitz, 2008; Gentile et al., 2011). For instance, active physical exploration of multisensory stimuli led to greater accuracy in an associative recognition task showing enhanced connectivity between sensory and motor cortices (Butler et al., 2011). Animal studies demonstrated that sensory feedback is crucial in motor learning. Monkeys with an ablated primary sensory hand area had no problems in executing a previously known task but were unable to learn new skills (Pavlides et al., 1993). Providing multisensory stimulation during goal-oriented action execution might help to establish sensorimotor contingencies (McGann, 2010). Muscle vibrations appear to influence the sensorimotor organization, whereas paired associative stimulation with TMS increases motor-evoked-potentials (Rosenkranz and Rothwell, 2006).

Of specific interest for rehabilitation is the integration of visual and proprioceptive information to perform movements. It has been shown that vision and proprioception are weighted differently at various stages during motor planning (Sober and Sabes, 2003), suggesting a target for multisensory manipulations. Concurrent haptic feedback during motor imagery appears to enhance the classification accuracy of brain-computer interfaces when decoding movement intention, indicating that it can aid in closing the sensorimotor loop (Gomez-Rodriguez et al., 2011). Multisensory stimulation training might help patients to recover from unimodal deficits, for instance, visual deficits or auditory localization deficits (Làdavas, 2008).

### Rhythmic Cueing

Neuroentrainment encompasses the study of the temporal relationship between the body’s movements and the rhythmic stimulation emerging from the environment. Any sensory modality (auditory, visual, tactile, or vestibular) can be used for entrainment (Ross and Balasubramaniam, 2014). To date, there is not much literature about visual entrainment, possibly because the auditory-motor synchronization appears to be mainly driving internal rhythmic movement control (Ross and Balasubramaniam, 2014). Hence, mainly auditory cues are used to synchronize movements to rhythmic patterns (Rossignol and Jones, 1976; Schaefer, 2014). Rhythmic patterns act like a template whose sequence can be anticipated (Nombela et al., 2013). The regularity detection and tempo tracking of rhythmic patterns increases the activity in motor network areas and cerebellum (Schaefer, 2014) and creates a mental representation of the rhythm, the so-called auditory model, which enables motor movements to anticipate the rhythmic pattern. The pooled evidence provided in the reviews by Grahn (2012) and Nombela et al. (2013) suggests that there are neuronal interactions between auditory and motor systems (Grahn, 2012; Nombela et al., 2013), and auditory-cued motor training can change their mutual structural connectivity (Moore et al., 2017). The auditory-motor action coupling relies on a subcortico-thalamic-cortical circuitry that can be activated through extrinsic cueing (Grahn, 2012; Nombela et al., 2013). Cerebellar patients cannot consciously perceive rhythm changes and show high variable motor responses. However, rhythmic synchronization, respectively, motor entrainment remains intact (Molinari et al., 2003), suggesting that the cerebellum might control the rhythmic auditory-motor synchronization by monitoring rhythmic patterns. Even without cueing, repetitive movements become periodic over time, as observed when analyzing gait patterns. The gait impairment observed in PD is ascribed to a deficiency of the internal timing ability that disturbs coordinated rhythmic locomotion, and which can be improved with rhythmical auditory stimulation (Thaut et al., 1996). Besides, rhythmic somatosensory cueing of stride frequency through vibrotactile stimulation at the wrist could improve qualitative walking performance in PD (van Wegen et al., 2006).

There is evidence that auditorily paced treadmill walking can improve gait coordination in stroke patients as well (Thaut and Abiru, 2010). Further, bilateral arm training with rhythmic auditory cueing enhances functional motor performance, which is maintained long-term (Whitall et al., 2000) and induces cortical and cerebellar changes (Luft et al., 2004). Meta-analyses found large effects that rhythmic auditory cueing improves walking velocity, cadence, and stride length (Yoo and Kim, 2016) and beneficial effects on improving upper limb impairment and function (Ghai, 2018) after stroke.

### Explicit Feedback/Knowledge of Results

KR has been defined as verbal, terminal and augmented feedback about goal achievement (Salmoni et al., 1984). Although the finding that extrinsic feedback can effectively create simple stimulus-response associations was brought forward by animal research in reinforcement learning, KR signifies more than just extrinsic rewards (Winstein, 1991; Schmidt and Lee, 2011). KR contributes to learning through cognitive processing, not through conditioning (Salmoni et al., 1984). KR is provided through explicit feedback. Explicit feedback is given on quantitative or qualitative task outcomes, e.g., correctness, exactness, success, or failure (Mazzoni and Krakauer, 2006; Subramanian et al., 2010; Schmidt and Lee, 2011). This feedback does not have to be verbal. For instance, when failing to reach for a target, the subject can hear unpleasant tones or see that the failed targets change color (Taylor et al., 2014). Also, explicit feedback about kinematic outcomes can be KR, e.g., playing back a recorded movement after execution. However, this feedback supports learning only if the movement features that led to the outcome are pointed out to the subject (Salmoni et al., 1984). Explicit feedback seems to activate explicit learning mechanisms and shows only subtle effects on implicit learning mechanisms (Taylor et al., 2014). While implicit learning appears to increase the cortical motor output maps of the involved movement initially, they return to baseline topography once the learned content can be explicitly declared. Possibly through explicit feedback a global motor plan is learned that is represented by higher-order neuronal networks, which influence the cortical sensorimotor representations differently (Pascual-Leone et al., 1994). Rewarding or punishing feedbacks appear to have dissociative effects on skilled motor learning. Punishment can speed up motor learning, whereas rewards ensure long-term retention (Abe et al., 2011; Galea et al., 2015). The reinforcement of positive outcomes appears to foster a success-driven learning system, which limits decay after learning, possibly by mobilizing the dopaminergic system (Wickens et al., 2003). Reward expectations modulate the activity of caudate neurons (striatal projection neurons), which receive reward-related information through the dopaminergic input from substantia nigra and spatial information through the cortico-striatal connection. Consequently, they modulate the inhibitory output of the basal ganglia, biasing attention to rewarded items. Either reward-driven activity of caudate neurons is a result of cerebral plasticity, or activity in the cerebral cortex is influenced by caudate neurons through the output nuclei of basal ganglia (Kawagoe et al., 1998). Dopamine has a gradual build-up and can persist for longer time courses; it might support long-term memory formation of motor actions (Abe et al., 2011).

KR has been used to reinforce adherence to CIMT (Taub et al., 1994). Meta-analyses often analyze KR together with KP under the umbrella term augmented feedback (van Dijk et al., 2005; Hayward et al., 2014). A meta-analysis analyzing different feedback types reported positive effects on motor function for KR (Molier et al., 2010). However, this evidence is based on one study (Eckhouse et al., 1990), whose intervention included other principles as well. It can, therefore, not be established whether KR is effective for motor recovery.

### Implicit Feedback/Knowledge of Performance

KP was defined as feedback given about movement execution in the form of verbal descriptions, demonstrations, or replays of recordings (Gentile, 1972). Advances in technology made it possible that KP can be delivered online, in an implicit manner and concurrent during movement execution, providing verbal or non-verbal feedback about ongoing intrinsic somatic processes and movement kinematics (Salmoni et al., 1984; Winstein, 1991). For instance, feedback in the form of sounds and colors can be given while trunk displacements surpass a threshold (Subramanian et al., 2007). Biofeedback uses physiological sources like electromyograms to provide patients with real-time visual or auditory signals about their motor activity (Huang et al., 2006). Ultimately arm movements can be visualized and augmented using VR representations (Ballester Rubio et al., 2015b; Ferreira dos Santos et al., 2016).

Implicit sensory feedback enhances learning from sensorimotor prediction errors, which for instance can aid the adaptation to unexpected perturbations (Shadmehr et al., 2010), possibly by contributing to implicit learning mechanisms (Taylor et al., 2014). Concurrent implicit feedback leads to lasting adaptations to visuomotor rotations, which are not (Hinder et al., 2008) or less observed (Taylor et al., 2014) when feedback about movement outcome, e.g., KR is given. Although KP appears to be beneficial during training, there is evidence that subjects can become dependent on it, showing inferior performance when feedback is removed (Ronsse et al., 2011). Ronsse et al. (2011) compared the effects of providing concurrent visual to concurrent auditory feedback during the acquisition of a bimanual movement pattern. The authors found that subjects that had obtained visual KP showed poorer performance during retention testing than subjects that were given auditory KP. During acquisition, the visual feedback increased the activity in vision/sensorimotor-specific areas, which was maintained during retention testing even in the absence of feedback. On the contrary, the concurrent auditory feedback reduced the activity in temporo-parieto-frontal areas and deactivated task-specific sensory areas during retention testing without feedback. These results suggest that subjects can become dependent on concurrent visual feedback, but not on concurrent auditory feedback because they rely on sensory processing areas that have become tuned to visual information during practice. The auditory feedback, on the other hand, might foster the formation of an internal controller, evidenced by the stronger activation of prefrontal areas. Alternatively, auditory feedback might promote reliance on proprioception and is consequently ignored during training (Ronsse et al., 2011). Results from cerebellar patients that were exposed to force-field learning tasks propose that the cerebellum may play an important role in using implicit information to correct and adapt motor commands to changed limb dynamics, and in forming internal controllers (Nezafat et al., 2001; Smith and Shadmehr, 2005; Tseng et al., 2007). In contrast to explicit error signals mediated through midbrain dopamine neurons in basal ganglia, implicit sensorimotor errors are possibly encoded by cerebellar climbing fibers and manifest in complex spikes in Purkinje cells during reaching tasks (Kitazawa et al., 1998). Computational modeling of adaptation to visuomotor rotations following concurrent visual feedback points to narrowly tuned neurons in the cortex that are driven by a prediction error that is computed by the cerebellum (Tanaka et al., 2009).

Stroke patients experienced a significant recovery in motor function and showed increased activation in the ipsilesional primary sensorimotor cortex after 4 weeks of training with a VR system that provided them with implicit feedback about their upper-limb movement (Jang et al., 2005). However, the system also included several other principles. In addition, the provision of KP has been shown to recover impaired movement patterns (Cirstea and Levin, 2007), to reduce learned non-use (Ballester Rubio et al., 2015b), and to lead to longer-lasting recovery effects (Subramanian et al., 2010). A meta-analysis found a beneficial effect for KP on motor function (Molier et al., 2010); however, the effect was based on two studies only.

### Modulate Effector Selection

In the acute stage after stroke, patients typically suppress the use of the affected limb due to pain, weakness, or malfunctioning (Taub and Uswatte, 2003). As a consequence, they are prone to overuse the non-paretic limb, and the resulting under-usage of the impaired limb can cause a loss of behavioral and neuronal function (Andrews and Stewart, 1979; Taub et al., 2006). Some authors argue that this compensation strategy, called learned non-use, emerges because the spontaneous use of the paretic limb does not cross a threshold level (Han et al., 2008). Although standard therapy focuses on improving the functionality of the impaired limb, the improvement does not transfer to increased use of the arm for ADLs (Smania et al., 2012; Kwakkel et al., 2015).

Of those therapeutic approaches that were successful in counteracting learned non-use CIMT is the most common and most successful one (Kwakkel et al., 2015). An fMRI study revealed changes in brain activity patterns due to paretic arm use in patients that underwent a 2 weeks CIMT program at home where the non-affected arm was constrained for 90% of the waking time. Increased grip strength in the affected limb correlated significantly with increased fMRI signal change in ipsilesional cortico-cerebellar areas (Johansen-Berg et al., 2002). However, a meta-analysis did not find a pooled effect that forcing the use of the paretic arm alone is effective (Hayward et al., 2014). Other approaches aimed at promoting paretic arm-use through positive reinforcement during bilateral arm training (Ballester Rubio et al., 2016) or through wearable devices (e.g., bracelets) that provide feedback about performance of ADLs (Ballester Rubio et al., 2015a).

### Action Observation/Embodied Practice

Action observation (Martens et al., 1976) gained increased attention after the discovery of mirror neurons (Rizzolatti and Sinigaglia, 2010): in monkeys, some neurons discharged not only when the animal executed a motor command but also when it observed another individual executing it. In humans, subjects who first observed other individuals performing a novel task performed better in the same task than control subjects that did not observe other individuals or observed a slightly different task (Mattar and Gribble, 2005). It is thought that in monkeys, as in humans, action observation relies on the frontoparietal network (Rizzolatti and Sinigaglia, 2010). Indeed, a meta-analysis showed that in humans, movement observation, as well as movement execution, recruits mainly the premotor and parietal areas. Movement observation, however, exclusively activated the visual cortex, whereas execution activated the primary motor cortex (Hardwick et al., 2018). Therefore, action observation might facilitate movement execution and motor learning by facilitating the excitability of the motor system (Mulder, 2007). Indeed, TMS during action observation elicited increased muscle activation patterns (Fadiga et al., 1995). For practical reasons, action observation could be especially beneficial for stroke patients with severe hemiparesis or complete paralysis. There is some clinical evidence that action observation therapy can reduce impairment and increase brain activation in the frontoparietal network and bilateral cerebellum (Ertelt et al., 2007).

Besides internalizing someone else’s movement, humans can also ascribe ownership and agency to body parts that do not pertain to them (Botvinick and Cohen, 1998). The discovery of rubber hand illusions (Botvinick and Cohen, 1998) led to insights about the mechanisms underlying agency. Both the sense of agency (Sato and Yasuda, 2005) and ownership are susceptible to manipulations (Slater et al., 2010), that have been used for therapeutic purposes, for instance, in mirror therapy (Ramachandran and Rogers-Ramachandran, 1996). Similar to action observation, mirror therapy appears to rely on the frontoparietal circuit (Harmsen et al., 2015), which is why its motor learning effects are partly explained by the same mechanisms (Hamzei et al., 2012). However, contrary to movement observation, mirror therapy robustly activates the primary motor cortex and visual processing areas ipsilateral to the mirrored movement. Also, mirror therapy seems to increase functional connectivity between cortical motor areas and to excite the neural connection between the two hemispheres (Hamzei et al., 2012; Arya, 2016). A meta-analysis attests mirror therapy a significant long-term effect on motor function, the ADLs, the reduction of pain and the reduction of visuospatial neglect (Thieme et al., 2012).

If the impairment of the limb impedes active movement, visual illusions could be presented to the patients to simulate movements with the paretic arm. The error-prediction mechanism driven by the cerebellum could be equally activated through the alternative representation (Fiorio et al., 2014). Possibly, the stronger the visual illusion, the more agency is ascribed to it, which could explain the difference in brain activation patterns between action observation and mirror therapy. The sense of agency seems to be important when learning from sensorimotor prediction errors (Tsakiris et al., 2007), respectively agency is reduced when prediction and outcome do not match (Sato and Yasuda, 2005). However, there is no consensus on the definition of ownership and agency, which makes their operationalization in clinical practice difficult.

### Mental Practice/Motor Imagery

Mental practice and motor imagery rely on the ability to simulate actions mentally without overt behavior, as summarized by the simulation theory (Jeannerod, 2001). Motor imagery can be seen as a mental rehearsal of future movements and motor plans (Naito et al., 2002; Schmidt and Lee, 2011), that can be beneficial for motor learning (Di Rienzo et al., 2016). However, actual physical practice shows superior effects on learning (Hird et al., 1991). A meta-analysis compared the brain areas that are active during mental imagery and movement execution. Both seem to recruit premotor areas, somatosensory cortex, and subcortical areas. Also, activation in the mid-cingulate cortex was found, with motor imagery activating more the anterior region that is linked to the cognitive aspects of motor control, whereas motor execution recruiting more the posterior region that is associated with basic motor functions. While motor imagery appears to activate more the parietal cortex, movement execution appears to recruit more classic sensorimotor regions like the primary motor cortex and cingulate motor areas (Hardwick et al., 2018). These findings are in line with studies showing that lesions in the frontoparietal system can diminish the ability of motor imagery (Johnson, 2000; Danckert et al., 2002). Motor imagery and physical practice also appear to induce similar learning-dependent brain changes (Di Rienzo et al., 2016). Not surprisingly, the activation pattern of motor imagery appears to be similar to the one identified in action observation and mirror therapy.

The learning effects of motor imagery and mental practice have been extensively studied in sports, whereas research regarding their clinical efficacy and efficiency is sparse and relatively recent (Mulder, 2007). However, motor imagery is thought to be advantageous for stroke recovery, especially for severely impaired patients (Mulder, 2007). Since patients retain the ability to imagine movements with the paretic limb, mental motor practice might facilitate functional reorganization (Johnson, 2000). A meta-analysis looking into the effectiveness of mental practice also found some trends for positive outcomes. However, pooled effects could not be estimated because only a few Class I studies exist, and their protocols, measurements, and interventions vary widely (Braun et al., 2006).

### Social Interaction

Social interaction has been defined as a behavior in which the participants’ actions are both a response to and a stimulus for the counterpart’s behavior (Rubin et al., 2006). Many ADL implicate social interaction, and a failure to perform them might lead to an undesired dependence on others (Lilja et al., 2003). The level of self-efficacy influences motor skill performance and learning, and in turn, is influenced by the appraisal or discouragement from others (Wulf et al., 2012). fMRI recordings of a subject experiencing a live social interaction revealed activations in areas commonly identified in the perception of social cues besides other regions involved in goal-directed and visual attention as well as reward processing (Redcay et al., 2010).

Animals that are allowed social interaction when recovering from an artery occlusion show higher functional improvement (Johansson and Ohlsson, 1996), increased recovery of behavior, and lower mortality, especially if the interaction partner was healthy (Venna et al., 2014). Including and investigating the impact of social interaction as part of the rehabilitation experience seems an important but missed opportunity. We found no study that was evaluating this specific aspect in a randomized controlled trial. One study evaluating enriched environments that included social interaction found positive results in terms of activity (Janssen et al., 2014).

## Discussion

This synthesis aimed at identifying a set of principles that should guide the design of effective neurorehabilitation protocols for post-stroke recovery. We identified 15 principles based on existing work on motor learning and recovery: massed practice/repetitive practice, spaced practice, dosage/duration, task-specific practice, task-oriented practice, variable practice, increasing difficulty, multisensory information, rhythmic cueing, explicit feedback/knowledge of results, implicit feedback/knowledge of performance, modulate effector selection, action observation/embodied practice, mental practice, and social interaction. Where possible, we identified the therapeutic and neurological effects of these principles from experimental work and clinical studies and commented on their limitations.

Our motivation for this analysis is twofold. Firstly, we are confident that the quality of evidence from clinical work and its interpretation would be enhanced if interventions are described along with the included principles. Reviews or meta-analyses with ambiguous effects often state that the included protocols remained vague on the exact experience provided to the patients, which makes the comparison and interpretation difficult (Veerbeek et al., 2014; Renton et al., 2017). By focusing solely on the ingredients of therapeutic interventions and compiling their current neuroscientific evidence, we aim to raise awareness of their importance. Also, this work might serve as a guide for clinicians and researchers to construct or identify the active ingredients in their interventions and to discover evidence currently missing. Secondly, we believe that there is a need to create a link between the principles of motor learning and their current operationalization in clinical studies and practice. We have identified several difficulties and shortcomings that do not aid in obtaining a common understanding of these principles and hence complicate the clinical investigation.

It seems that many principles are poorly operationalized in clinical trials. For instance, when massed practice is investigated, the repetitions performed within a session and during the treatment duration are rarely quantified (Lang et al., 2007) such that recovery effects due exclusively to repetition cannot be singled out. Also, the clinical research of spaced practice and dosage/duration would benefit if the parameters were quantified in a standardized way. Particularly dosage should be explicitly described in treatment minutes per session in order to be able to establish a dose-response due to training (Dobkin, 2005). Furthermore, dosage/duration should not be equated with intensity since the intensiveness of training cannot be estimated through treatment minutes only (Billinger, 2015). Intensity should be an independent principle that needs to be investigated separately. Task-specific and goal-oriented practice appear to be often used interchangeably (Winstein et al., 2014; Fu et al., 2015; Yue et al., 2017) although their training target is different. While task-specific practice focuses on the acquisition of a specific skill (Keetch et al., 2005) for ADL, goal-oriented practice permits the use of any movement or skill that is deemed suitable to achieve the goal (Horak, 1991), fostering the exploration of alternative movement patterns. Variability appears to be included inherently in many protocols (Darekar et al., 2015), possibly because it renders the training less repetitive and, therefore, less boring (Birkenmeier et al., 2010), which could counteract low adherence. However, this link has not been explicitly studied. Increasing the difficulty during practice is part of many intervention protocols as well; however, personalizing the difficulty level in order to provide training at the optimal challenge point seems to be rarely addressed. Concerning multisensory integration, it would be interesting to explore whether the presence of more than two sensory stimulations could enhance learning (Sánchez et al., 2013). Similarly, rhythmic entrainment could be extended with protocols exploring if visual or haptic entrainment might aid recovery of impaired movements (Penhune et al., 1998). Explicit feedback and implicit feedback are often investigated together under the umbrella term of augmented feedback, as evidenced by the sourced meta-analysis and clinical studies (Molier et al., 2010). However, their aim and the neuronal mechanisms that they appear to stimulate are different. While explicit feedback provides terminal feedback about movement outcome, implicit feedback provides concurrent error-signals during movement execution fostering possibly different learning mechanisms. Meta-analyses also appear to interpret the sensory modality of the feedback, e.g., if it is visual, auditory or haptic as a feedback type. However, the sensory modality is a separate layer that is added to feedback. Explicit feedback, as well as implicit feedback, can be unisensory or multisensory. Action observation and mirror-therapy appear to be well studied therapeutic ingredients, whereas mental practice is only addressed in a few studies, and social interaction remains unexplored territory so far. If the principles would be better operationalized, it would not only help to identify their contribution to the recovery of motor functions, but also other learning outcomes such as cognitive or language improvements.

The neuronal changes found within each principle allow us to draw some general conclusions for the advancement of neurorehabilitation. While some principles appear to modulate more specific brain areas (massed practice, dosage, variable practice, task-specific practice, modulate effector selection, multisensory stimulation) within the motor areas of the cortex others appear to recruit or rely more on networks of brain regions (goal-oriented practice, increasing difficulty, action observation, motor imagery, mirror therapy, rhythmic cueing, implicit feedback/knowledge of results, social interaction). An effective rehabilitation approach should thus incorporate principles of both types in order to counteract neuronal degradation and promote improvement. Firstly, a training that addresses only a limited subset of the neuronal circuitry underlying a general function might limit transfer to other behaviors that depend on the same circuitry (Kleim and Jones, 2008). Secondly, not all principles are equally applicable to all patients. Some principles might be more beneficial early after stroke, whereas others benefit patients with less severe damage. Spontaneous biological recovery and activity-dependent plasticity appear to interact differently at different stages after stroke, which, aside from other factors like severity, predicts recovery (Reinkensmeyer et al., 2016; Hylin et al., 2017). It seems that in acute patients the sensorimotor cortex activity is highly abnormal, and the normalization in activity patterns is linked to better recovery (Schaechter, 2004). Principles like task-oriented practice that promote localized changes, might therefore be more beneficial at the acute stage after stroke (Schaechter, 2004), whereas therapies like CIMT, where the forced use of the impaired limb is paired with increasing difficulty and further principles, have been shown to be more suitable at later stages after stroke and for less impaired patients (Dromerick et al., 2009). More severely impaired patients, on the other hand, might benefit from action observation, mirror therapy and motor imagery (Dohle et al., 2009; Sun et al., 2013). Future studies will show the optimal combinations of principles that stimulate plasticity in a way that learning of preexisting or novel functions is enhanced.

We are aware that the view proposed here is strongly influenced by knowledge mainly derived from clinical work with hemiparetic stroke patients. However, the literature indicates that other diseases, for instance, PD (Rossiter et al., 2014) or Alzheimer’s disease (Kalaria, 2002), show similar cognitive, functional, and neuronal alterations even though they may have different pathologies. Therefore, these principles of neurorehabilitation could be potentially applied beyond the field of stroke. As our main goal was to provide a synthesis that is informative and practical, in-depth analysis of each principle and its neurological underpinnings lie outside of the scope of this work. In future work, we will unify the principles addressed here in a theoretical framework to show how each of them contributes to the restoration of sensorimotor contingencies (Verschure, 2011).

In summary, our review provides a synthesis of effective therapeutic ingredients that could be beneficial in aiding recovery after stroke. We hope that future work will extend the evidence presented here by implementing and investigating the principles of neurorehabilitation in novel rehabilitation protocols for stroke and other patient populations.

## Author Contributions

MM, BB, and PV contributed to the design of the review. MM wrote the first draft of the manuscript and performed the computerized search. All authors contributed to the manuscript revision, read and approved the submitted version.

## Conflict of Interest

PV is the founder and interim CEO of Eodyne Systems S.L., which aims at bringing scientifically validated neurorehabilitation technology to society. The remaining authors declare that the research was conducted in the absence of any commercial or financial relationships that could be construed as a potential conflict of interest.

## Abbreviations

ADLs: activities of daily living
CIMT: constraint-induced movement therapy
fMRI: functional magnetic resonance imaging
KR: knowledge of results
KP: knowledge of performance
LTP: long-term potentiation
PD: Parkinson’s disease
TMS: transcranial magnetic stimulation
VR: virtual reality.
